# Single vs. double purse-string anastomosis during laparoscopic low anterior rectal resection (SINGLE–DOUBLE trial): study protocol for a randomized controlled trial

**DOI:** 10.1186/s13063-019-3411-7

**Published:** 2019-05-28

**Authors:** Xiaolan You, Jian Wu, Yuanjie Wang, Qinghong Liu, Dehu Chen, Xiaojun Zhao, Yan Zhou, Xiaoqing Wu, Daorong Wang

**Affiliations:** 1 Department of Gastrointestinal Surgery, The Hospital Affiliated to Medical School of Yangzhou University (Taizhou People’s Hospital), 366 Taihu Road. Hailing District, Taizhou, Jiangsu 225300 China; 2 Department of Gastrointestinal Surgery, Clinical Medical College of Yangzhou University (Subei People’s Hospital of Jiangsu Province), 98 West Nantong Road. Hanjiang District, Yangzhou, Jiangsu 225003 China

**Keywords:** Rectal cancer, Laparoscopy, Total mesorectal excision, Double purse anastomosis, Single purse anastomosis

## Abstract

**Background:**

An inappropriate anastomosis method during laparoscopic anterior rectal resection can increase the risk of anastomotic complications and affect surgical, economic, and oncological outcomes. The aim of this study is to compare the incidence of anastomotic complications and the surgical, economic, and oncological outcomes following single versus double purse-string anastomosis during laparoscopic total mesorectal excision (TME) for low rectal cancer.

**Methods/design:**

This randomized controlled trial (the SINGLE–DOUBLE study) will randomly assign middle and low rectal adenocarcinoma patients to receive either single or double purse-string anastomosis during laparoscopic low anterior rectal resection. Patients will be eligible for inclusion only if they (1) have adenocarcinoma confirmed by preoperative colonoscopy and biopsy, (2) have a tumor situated less than 12 cm from the anal verge, (3) do not have the anal sphincter involved, and (4) do not have distant metastases. The primary endpoint measure will be the incidence of anastomotic complications (leakage, narrowing, and bleeding). The secondary endpoints will be surgical, economic, and oncological outcomes. A total of 500 patients will be enrolled in the study. Sample size calculation was based on previous reports and our retrospective analysis.

**Discussion:**

This randomized single-center controlled trial is expected to demonstrate which anastomosis method (single or double purse-string anastomosis) is better for reducing complications and improving prognosis in rectal cancer patients undergoing laparoscopic TME for low or middle rectal cancer.

**Trial registration:**

Registration number: ChiCTR1800016116. Protocol Registration Receipt: May 13, 2018.

**Electronic supplementary material:**

The online version of this article (10.1186/s13063-019-3411-7) contains supplementary material, which is available to authorized users.

## Background

Colorectal cancer is the third most common malignancy in both sexes worldwide [1]. Almost one third of colorectal cancers are located in the rectum and these are reported to be associated with worse prognosis [2–4]. Total mesorectal excision (TME) has been the gold standard treatment for locally advanced rectal cancer ever since Heald et al. first described it in 1982 [5]. With TME, it is possible to radically excise the cancer with relatively little damage to surrounding tissues. It maximizes functional outcomes, greatly reduces the risk of local recurrence, and promotes survival [6].

With the rapid advances in laparoscopic technology over the past 20 years, the advantages of laparoscopic surgery have become more and more obvious: it provides better cosmetic outcome, causes less tissue trauma and postoperative pain, and decreases the risks of wound infection, postoperative respiratory complications, and incisional hernia [7]. Therefore, laparoscopic TME has replaced open TME as the gold standard for rectal cancer surgery [8–10]. However, some controversies persist about which type of anastomosis is best for reducing the incidence of anastomotic complications [11–13], especially during laparoscopic TME for low rectal cancer.

In 1979, Ravitch and Steichen [14] first proposed the end-to-end anastomosis for low rectal cancer. This procedure was improved by the use of a straight-line stapler (TA-55) to close the distal rectal segment, an end-to-end colorectal anastomosis performed using double staplers, and a tubular stapler inserted through a linear stapler via the anus. According to Griffen et al. [15], this kind of anastomosis has several advantages: (1) it avoids the technical difficulties involved in distal rectal suturing, (2) it makes anus-preserving surgery in lower rectal cancer technically feasible, (3) it reduces the possibility of contamination because the distal rectal segment is not opened, and (4) it makes it possible to connect intestinal segments of different diameters (the wider rectal ampulla and the narrower sigmoid colon) and thus reduces the complications of anastomosis. This anastomosis method is now widely applied, and laparoscopic linear stapling is used to close rectal stump. However, with its increasing use, some disadvantages are becoming apparent. This kind of anastomosis is usually a combination of an end-to-end anastomosis and an end-to-side anastomosis, which results in an incomplete internal wall of the anastomosis and increased scope of anastomosis, thus increasing the risk of bleeding. In addition, there is often poor blood supply to the anastomotic triangle, which further increases the risk of anastomotic leakage. Generally, purse-string sutures are used to close the proximal and distal rectum end-to-end colorectal anastomosis by a single stapler can provide a smooth inner wall at the anastomosis and reduce the length of the anastomosis. Double purse-string anastomosis by a single stapler can provide a smooth inner wall at the anastomosis and reduce the length of the anastomosis. However, the procedure is difficult to perform within the confines of the pelvic space.

This randomized controlled trial aims to determine whether there is a difference in the incidence of anastomotic complications (leakage, narrowing, and bleeding) and various surgical, economic, and oncological outcomes between patients receiving single purse-string anastomosis versus double purse-string anastomosis during laparoscopic TME for low rectal adenocarcinoma.

## Methods/design

### Study design

**Fig. 1:**
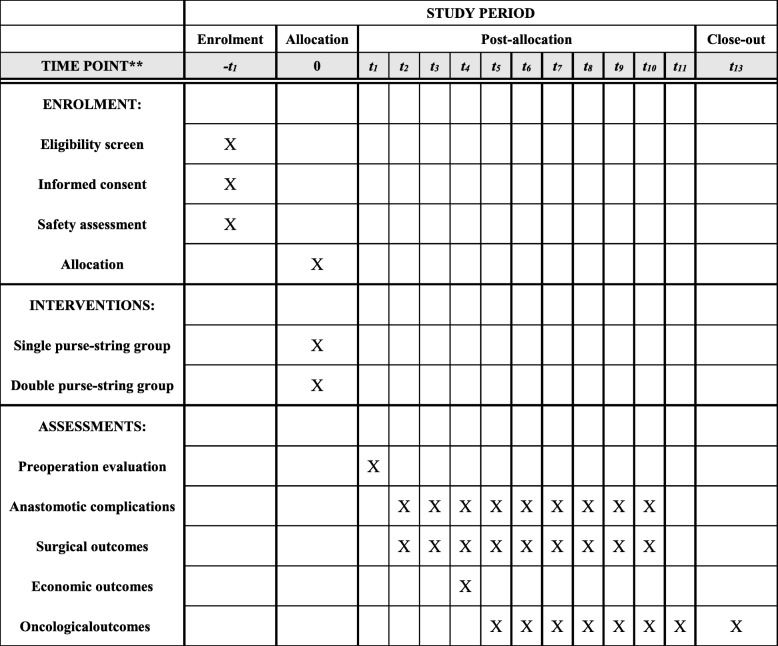
SPIRIT (Standard Protocol Items: Recommendations for Interventional Trials) diagram for schedule of enrollment, interventions, and assessments. Safety assessment includes routine blood, routine stool, routine urine, liver function, renal function, coagulation test, syphilis, hepatitis B virus (HBV), hepatitis C virus (HCV), HIV, and electrocardiogram. *Abbreviations*: *t1* 1 day after allocation, *t2* 1 day after surgery, *t3* 1 week after surgery, *t4* discharge from hospital, *t5* 1 month after surgery, *t6* 6 month after surgery, *t7* 1 year after surgery, *t8* 1 and a half years after surgery, *t9* 2 years after surgery, *t10* 3 years after surgery, *t11* 4 years after surgery, *t12* 5 years after surgery

**Fig. 2:**
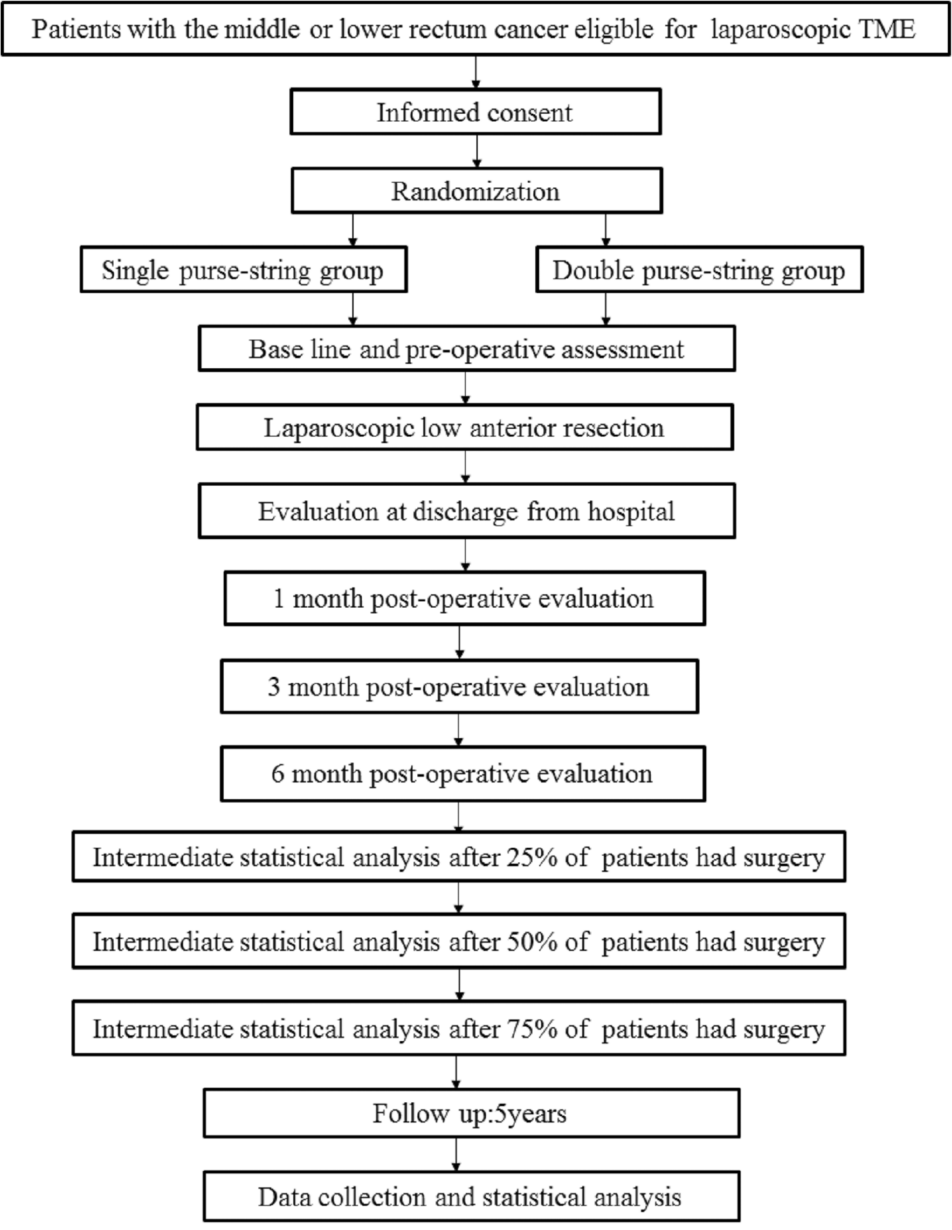
Study flowchart. TME: Total mesorectal excision

This prospective single-center randomized clinical trial to be conducted at the Taizhou People’s Hospital of Jiangsu Province began in September 2018 and is expected to end in 2023. Patients with diagnosed rectal cancer and meeting the entry criteria will be recruited and randomly assigned to receive either single purse-string anastomosis or double purse-string anastomosis. All operations will be performed by the same group of surgeons. Baseline demographic and clinical features, surgical indices, pathological findings, health economic indices, postoperative complications, local recurrence, distant metastasis, disease-free survival, and overall survival will be compared between the two groups. Figure 1 shows the schedule of enrollment and interventions, and assessment is provided in the Standard Protocol Items: Recommendations for Interventional Trials (SPIRIT). Figure 2 shows the study flow. The SPIRIT checklist is presented in Additional file 1.

### Study population

The study population will comprise patients with rectal cancer. The inclusion criteria will be (1) low or middle rectal adenocarcinoma, in which the tumor is situated less than 12 cm from the anal verge, (2) diagnosis confirmed by preoperative colonoscopy and biopsy, (3) patient eligible for low anterior rectal resection with laparoscopic TME, and (4) willingness for complete 5-year clinicopathological follow-up. Exclusion criteria will be (1) low rectal adenocarcinoma with anal sphincter involvement, (2) presence of distant metastases, (3) radical resection not performed because of local invasion, (4) presence of other synchronous malignancy or serious disease, and (5) surgery performed as an emergency procedure.

### Primary and secondary endpoints

The primary endpoint will be the incidence of anastomotic complications (i.e., anastomotic leakage, anastomotic narrowing, and anastomotic bleeding). Anastomotic leakage will include clinical and subclinical anastomotic leakage. The diagnosis of clinical anastomotic leakage is based on the clinical manifestations and the extravasation of contrast medium after rectal enema showed on the computed tomography (CT) scan. However, the subclinical anastomotic leakage has only extravasation of contrast medium [16]. Anastomotic narrowing will include both clinically evident narrowing and subclinical narrowing proved by endoscopic examination at 6 months after surgery. On endoscopy, anastomotic stricture will be diagnosed when the diameter of the anastomotic stoma is less than 11 mm [17]. Anastomotic bleeding will be determined as early postoperative anastomotic bleeding or delayed bleeding, and the bleeding of rectum within the first four weeks after rectal anastomosis will be considered as the early postoperative anastomotic bleeding [18].

The secondary endpoints will be the surgical, economic, and oncological outcomes. Indices of surgical outcome will include surgery duration, intraoperative blood loss, anus retention ratio, ileostomy rate, the number of patients with changed method of anastomosis, bowel function recovery time, mean size of tumor, proximal margin status, distal margin status, number of lymph nodes harvested, and postoperative defecation and genitourinary dysfunction. Economic evaluation includes direct and indirect costs, direct costs will include duration of hospital stay and medical costs, and subsequent direct cost-effectiveness analysis will be performed. Indices of oncological outcome will include tumor-node-metastasis (TNM) stage, histology stage, local recurrence, distance metastasis, disease-free survival, and overall survival.

### Randomization and blinding

Patients meeting the eligibility criteria will be randomly assigned (1:1) to receive either single purse-string anastomosis or double purse-string anastomosis. Randomization will be performed by using TenAlea software (ALEA Clinical, Abcoude, The Netherlands).

Given the obviously different characteristics of the two types of anastomoses, blinding of the surgeons will not be possible. However, patients, nursing staff, and statisticians will be blinded to treatment allocation during data collection and analysis.

### Preoperative evaluation

Data will be collected on age, gender, body mass index, American Society of Anesthesiologists (ASA) class, plasma albumin, hemoglobin, previous abdominal or pelvic surgery, tumor size, distance of tumor from anal margin, and tumor pathological differentiation. All patients will be assessed by the Low Anterior Resection Syndrome (LARS) score [19], Fecal Incontinence Quality of Life Scale (FIQL) [20], International Index of Erectile Function (IIEF) [21], International Consultation on Incontinence Questionnaire (ICIQ) [22, 23], and Female Sexual Function Index (FSFI) [24].

### Preoperative preparation

All patients will undergo the enhanced magnetic resonance imaging scan of the pelvis for preoperative staging, enhanced CT scan of the abdomen to exclude distant metastasis, chest radiography, cardiac ultrasonography, electrocardiogram, and other examinations to exclude surgical contraindications. Preoperatively, patients will receive only fluids for 24 h and orally take 2000 mL of 6.8% polyethylene glycol-electrolyte solution for bowel preparation.

### Surgery

The following steps of laparoscopic anterior rectal resection with TME will be common to all patients. The sigmoid mesentery will be incised by using an ultrasonic knife through the middle approach, and the Toldt space will be entered. The peritoneum will be incised toward the duodenojejunal angle (Treitz) until the root of the mesentery. Then the left retroperitoneal space and presacral space will be expanded. The left colic artery will be identified and preserved, and the inferior mesenteric artery will be ligated. Dissection is then continued windowing Toldt’s space, divide rectal to the pelvic side until the levator ani muscle plane. The left ureter, submesenteric plexus, genitofemoral nerve, hypogastric nerves, common iliac veins, and gonadal vessels will be identified and preserved. The left part of the gastrocolic ligament will be divided, and the left part of the transverse mesocolon will be opened. The splenocolic and phrenocolic attachments will be divided to free the left colonic angle completely. The length of rectum to be resected (>2 cm distal to the margin of tumor) will be exposed, and the proximal sigmoid mesentery will be cut to the pre-resect site (15 cm from the superior margin of the tumor).

### Single purse-string anastomosis

In the single purse-string group, the distal rectal will be occluded by long-mouth atraumatic forceps, the perineal team will slowly dilate the anus and control the degree of the relaxation, and the rectum will be washed with physiological saline. Laparoscopic transection of the distal rectum will be performed by using a linear cutting stapler. A 5-cm longitudinal incision will be made above the pubis to enter the abdomen. The proximal sigmoid colon will be cut under direct vision, and the end will be closed with purse-string suture. The surgical specimen will be removed. Finally, a tubular stapler will be inserted via the anus, and end-to-end anastomosis of the sigmoid and the rectal stump will be performed.

### Double purse-string anastomosis

In the double purse-string group, a 5-cm long longitudinal suprapubic incision will be used to enter the abdomen after the intestinal canal has been laparoscopically exposed. Then cut the proximal sigmoid colon, the end closed with purse string suture and embed the bottom nail base of tubular stapler. The distal rectum will be occluded with large right-angled forceps under direct vision, after which the perineal team will slowly dilate the anus and wash the rectum with physiological saline. With traction applied to the distal rectum, purse forceps will be applied just distal to the right-angled forceps. Purse suture will be applied and the rectal stump cut. A tubular stapler is introduced into the rectal segment with the center rod retracted within the cartridge, then unscrew the rod of bottom nail to back-out the center rod and tighten up the purse. The trocar is removed and the anvil shaft is inserted into the rod, end-to-end anastomosis of the sigmoid and rectal stump will be performed.

### Postoperative evaluation

A postoperative schedule of evaluation is shown in Fig. 2. At the early postoperative evaluation, we will assess surgical indices (surgery duration, intraoperative blood loss, anus retention ratio, ileostomy rate, the number of patients with changed method of anastomosis, and bowel function recovery time), economic indices (hospitalization days and medical expenses), pathology findings (mean size of tumor, proximal margin, distal margin, number of lymph nodes harvested, TNM stage, and histology stage), and early anastomotic complications (leakage and bleeding). At 1 month after surgery, colonoscopy will be performed to evaluate the anastomosis (leakage, stricture, and signs of ischemia). The LARS score [19], FIQL [20], the IIEF [21], ICIQ [22, 23], and FSFI [24] will be used to investigate defection and genitourinary function. At 3 months after surgery, colonoscopy will be performed to look for anastomosis leakage and stricture, and genitourinary function will be assessed. At 6 months after surgery, anastomosis leakage, anastomosis stricture, and defecation and genitourinary function will be assessed again. One year after 25%, 50%, and 75% of patients had surgery, intermediate statistical analysis will be carried out by the staffs of the medical quality control department and will be reviewed by the staffs to evaluate whether the expected ratio of complication is respected and whether any of the techniques is harmful for the patients before the end of the inclusion period. Oncological follow-up will be carried out for 5 years in accordance with the 2015 National Comprehensive Cancer Network guidelines for rectal cancer surveillance (http://www.nccn.org/professionals/physician_gls/pdf/rectal.pdf) and will include periodic assessment for local recurrence, metastasis, disease-free survival, and overall survival.

### Power calculation and sample size

We calculated the sample size by using the calculator available at powerandsamplesize.com/Calculators/Compare-2-Proportions/2-Sample-Equality. A comprehensive analysis of a number of studies showed that the incidence of postoperative anastomotic leakage was 8.3% (154/1861) after single purse-string anastomosis of laparoscopic low anterior resection. The retrospective analysis showed that the incidence of leakage was 1.8% (2/108) in the patients with double purse-string anastomosis of laparoscopic low anterior resection in our hospital. The power computation is aimed at detecting a minimum clinically meaningful odds ratio of 0.203 (i.e., a reduction of 79.7% anastomotic leakage in double purse string as compared with single purse string). To detect this difference with α = 0.05 and power of 1 − β = 0.80, 174 per group would be necessary. Given an estimated dropout rate of 20%, the minimum required sample size would be 436 patients. We plan to enroll a total of 500 patients (250 per group).

### Statistical analysis

Statistical Package for the Social Sciences (SPSS) 16.0 (SPSS Inc., Chicago, IL, USA) will be used for statistical analysis. Continuous variables will be summarized as mean (± standard error) and will be compared between groups by using the Student’s *t* test. Anastomotic leakage, anastomotic narrowing, anastomotic bleeding, and other categorical variables will be summarized as percentages and compared by using the chi-squared test. The odds ratio for having anastomotic complications will be performed with the logistic regression model. The Kaplan–Meier method will be used to perform the survival analysis, and the log-rank test will be applied to analyze differences between groups. Univariate analysis will be performed to identify variables associated with prognosis. Cox regression analysis will be used to identify independent predictors of prognosis. Statistical significance will be at *P* ≤0.05.

### Data collection and monitoring

Patients will fill out questionnaires both before and after surgery. The operation will be performed by the same surgical team, and colonoscopies will be performed by the same senior endoscopic examiner. The study coordinators will be regularly contacted through monthly meetings. Two research fellows will enter the data into a Microsoft Access database every day.

## Discussion

This study aimed to determine which method of anastomosis (single purse string versus double purse string) is better for preventing complications and improving outcomes after laparoscopic TME for rectal cancer.

Anastomotic leakage is the most common complication in laparoscopic TME and is the best measure of the success of rectal reconstruction. Poor blood supply to the anastomosis increases the risk of developing anastomotic leakage [25]. In patients receiving single purse-string anastomosis, the triangle region and the “cat’s ear” on either side of the anastomosis have poor blood supply and therefore are particularly vulnerable sites [26]. The double purse-string anastomosis eliminates the triangle region and the residual horns, but whether it reduces the risk of anastomotic leakage is not known. In addition, during single-string anastomosis, it is difficult to insert the line stapler to close the rectal stump because of the narrow pelvic space, and multiple applications of the linear stapler may be necessary. The increased number of linear stapler firings increases the risk of developing anastomotic leakage [27].

The incidence of anastomotic bleeding after anterior rectal resection is reported to be 0.4% [28]. The risk factors for anastomotic bleeding have not yet been fully elucidated and so it is difficult to predict or prevent anastomotic bleeding. Whether the double purse-string technique can reduce the risk of secondary bleeding by shortening the length of the anastomosis needs to be explored.

When the anastomosis in low rectal TME is created with the use of a mechanical stapler, the rate of anastomotic stricture is reported to be as high as 20% [29]. Studies suggest that poor epithelial bridging of the exposed serous layer and scar formation may be a reason for the increased incidence of stricture after stapler anastomosis. Anastomotic leakage and anastomotic ischemia are also important causes of anastomotic stricture [29]. A significantly lower incidence of anastomotic complications in one of the groups in this study could indicate that the anastomotic method plays a key role.

Surgical, economic, and oncological outcomes are also important indices of the feasibility and success of an operation. This study seeks to establish whether double purse-string anastomosis has any significant impact on surgical, economic, and oncological outcomes in patients undergoing low anterior rectal resection. Increase in the rate of anal preservation and reduction of defecation and genitourinary dysfunction following laparoscopic low anterior rectal resection will be an indication that the technique could improve the quality of life of patients [30]. Pre- and post-operative assessments of defecation and genitourinary function will allow complete evaluation of the impact of the operation. This randomized controlled trial will also avoid the biases inherent in retrospective evaluations. We expect that the double purse-string group will present fewer anastomotic complications, shorter hospital stays, and reduced medical costs.

## Trial status

The study is not yet open for participant recruitment.

## Additional file

Additional file 1: SPIRIT (Standard Protocol Items: Recommendations for Interventional Trials) 2013 Checklist. (DOC 123 kb)

## Abbreviations

CT: Computed tomography
FIQL: Fecal Incontinence Quality of Life Scale
FSFI: Female Sexual Function Index
ICIQ: International Consultation on Incontinence Questionnaire
IIEF: International Index of Erectile Function
LARS: Low Anterior Resection Syndrome
SPIRIT: Standard Protocol Items: Recommendations for Interventional Trials
TME: Total mesorectal excision
TNM: Tumor-node-metastasis
